# Disparities in Utilization and Delivery Outcomes for Women with Perinatal Mood and Anxiety Disorders

**DOI:** 10.20900/jpbs.20240003

**Published:** 2024-04-30

**Authors:** Kara Zivin, Anna Courant

**Affiliations:** 1 Department of Psychiatry, University of Michigan Medical School, Ann Arbor, MI 48109, USA; 2 Program on Women’s Healthcare Effectiveness Research, Department of Obstetrics and Gynecology, University of Michigan Medical School, Ann Arbor, MI 48109, USA; 3 Department of Obstetrics and Gynecology, University of Michigan, Ann Arbor, MI 48109, USA; 4 Institute for Healthcare Policy and Innovation, University of Michigan, Ann Arbor, MI 48109, USA; 5 Department of Health Management and Policy, University of Michigan School of Public Health, Ann Arbor MI 48109, USA; 6 VA Ann Arbor Healthcare System, Ann Arbor MI 48105, USA

**Keywords:** PRAMS, perinatal mood and anxiety disorders, perinatal depression, perinatal anxiety, perinatal mental health

## Abstract

Perinatal mood and anxiety disorders (PMAD), which include depression and/or anxiety in the year before and/or after delivery, are common complications of pregnancy, affecting up to one in four perinatal individuals, with costs of over $15 billion per year in the US. In this paper, we provide an overview of the disparities in utilization and delivery outcomes for individuals with perinatal mood and anxiety disorders in the US. In addition, we discuss the current US screening and treatment guidelines as well as the high societal costs of illness of PMAD for both perinatal individuals and children. Finally, we outline opportunities for quality improvement of PMAD care in the US, including leveraging increased engagement with healthcare system during prenatal care, working toward a more cohesive national strategy to address PMAD, leaning into evidence-based policymaking through collaboration with a panel of experts, and generating state-level profiles focused on PMAD.

## INTRODUCTION

Perinatal mood and anxiety disorders (PMAD), which include depression and/or anxiety in the year before and/or after delivery, are common complications of pregnancy, affecting up to one in four perinatal individuals, with costs of over $15 billion per year in the US [1–4]. PMAD are associated with adverse birth outcomes and are the leading causes of maternal mortality (i.e., postpartum suicides) [5–7]. Mental health (MH) conditions such as PMAD are leading causes of pregnancy-related deaths [8], which are rising in the US [9]. Perinatal individuals with PMAD are at higher risk for preterm birth, cesarean delivery, and severe maternal morbidity (SMM) [10,11]. PMAD can affect perinatal individuals, babies, and families beyond the perinatal period, with lasting clinical and economic impacts [12]. PMAD treatment can improve maternal and neonatal health outcomes [13–17], yet treatment is rare; <10% of individuals with PMAD receive treatment, and <5% reach remission [18].

In this paper, we provide an overview of the disparities in utilization and delivery outcomes for individuals with PMAD in the US. Our “Introduction” section provides background information by focusing on disparities, recent findings, and policy, while the “Discussion” section delves into outcomes, screening, costs, and opportunities for quality improvement. Specifically, we discuss the current US screening and treatment guidelines as well as the high societal costs of illness of PMAD for both perinatal individuals and children. We also outline opportunities for quality improvement of PMAD care in the US, including leveraging increased engagement with healthcare system during prenatal care, working toward a more cohesive national strategy to address PMAD, leaning into evidence-based policymaking through collaboration with a panel of experts, and generating state-level profiles focused on PMAD.

Black, Asian, Hispanic, rural, and low income individuals with PMAD have more severe episodes, and lower treatment initiation, engagement, adherence, and retention rates than White, urban, higher income individuals [19–24]. Previous research identified other pregnancy-related racial/ethnic health disparities as well as disparities in maternal mortality and morbidity.

With regard to maternal mortality, for example, researchers found that non-Hispanic Black and non-Hispanic American Indian/Alaska Native women experienced higher pregnancy-related mortality than did all other racial/ethnic groups [25,26]. In another analysis, non-Hispanic Black women had the maternal mortality rate 3.55 times than non-Hispanic White women [27]. The COVID-19 pandemic exacerbated these maternal mortality rates and racial disparities among non-Hispanic Black and Hispanic women, who experienced higher COVID-19 related deaths than White women [26].

With regard to maternal health disparities, maternal racial/ethnic health disparities persist even after controlling for education level and income, indicating the need to address structural factors such as racism and discrimination within the healthcare system [26]. Respectful maternity care represents an example of one framework for improving these outcomes in Black women by addressing disrespectful care during childbirth that may contribute to these disparities through recognition of multiple levels of influence on maternity care, from disrespect and abuse to integration of rights-based approaches rooted in reproductive justice, human rights, social justice, and antiracism [28]. The US government has also outlined federal-level approaches to reducing racial/ethnic disparities in maternal health outcomes, including the 2022 White House Blueprint for Addressing the Maternal Mental Health Crisis; these approaches are multi-faceted and incorporate improving access to quality maternity care, expanding medical insurance coverage, and standardization of care, among others [29,30].

In this context, our team undertook a series of population-level analyses aiming to learn more about patterns of care and outcomes, including health disparities as well as disparities in maternal mortality and morbidity. With regard to maternal mortality, when we analyzed trends in suicidality among childbearing individuals from 2006 to 2017, we found that despite substantially increasing prevalence of suicidal ideation and self-harm occurring in the year preceding or following birth for all perinatal individuals, non-Hispanic Black individuals, those with low-income, and younger individuals had greater escalations, as did those with comorbid anxiety, depression, or other serious mental illness than other racial/ethnic groups, higher income individuals, and older individuals [31]. In another analysis spanning 2008 to 2018, our team also found that Black individuals experienced the sharpest proportional increases of suicidal ideation diagnosis during pregnancy compared to all groups [32].

With regard to other maternal health disparities, we found that Black individuals experienced the highest rates of delivery-related severe maternal morbidity when compared to other racial/ethnic groups during hospitalization for birth, hospital discharge to 42 days postpartum, and 43 to 365 days postpartum [33]. We also found that rates of perinatal psychotherapy utilization increased during 2008 to 2020 across all racial/ethnic groups, with Asian individuals experiencing the most pronounced increase in use [34].

With regard to infant outcomes, we found that pregnant people with antenatal anxiety or depression and Black perinatal people had a higher likelihood of delivering infants with adverse birth outcomes [35]. We also found that postpartum women whose infants had NICU hospitalization had greater odds of maternal anxiety and/or depression diagnoses in the year postpartum; however, although we found that a higher proportion of Black, Hispanic, and Asian infants experienced NICU admission, the birthing parents of these infants had significantly lower odds of receiving such a diagnosis when compared to White women, which may indicate a racial disparity in such diagnoses in this population [36].

Considering these contributions, we also recognize that no comprehensive data source documents the magnitude, predictors, and variation in disparities by race and ethnicity, socioeconomic status, and geography in healthcare utilization or obstetric delivery outcomes among individuals with PMAD. Almost no evidence documents the impact of community characteristics on these outcomes. It remains critical to understand both patient and community factors associated with ineffective or absent PMAD treatment to effectively target resources, clinical care, and policies [37,38]. No overarching national plan outlines how best to address PMAD, overall, or within high-risk subgroups, leaving each state to determine how best to care for these individuals.

Another approach is to focus efforts at the state level. State policy can influence access to PMAD treatment since each state determines individually how to address PMAD. Our team of researchers is using data from publicly and privately insured individuals with PMAD, state survey data with input from a panel of perinatal MH services and policy experts. We study this data to identify patient-level clinical characteristics associated with MH and overall perinatal healthcare utilization and delivery-related outcomes among individuals with diagnosed PMAD, establish accurate national and state-level estimates of disparities in care patterns and outcomes, and determine the contributions of community-level characteristics to perinatal utilization and delivery outcomes among individuals with PMAD. Given the dearth of research on disparities in addressing PMAD and associated utilization and delivery outcomes as well as high, intergenerational costs for birthing parent and baby of ineffectively managed PMAD, we hope that our innovative, large-scale investigation will provide evidence necessary for future policymaking and clinical interventions tailored to population needs. This work has become even more urgent in light of the large increases in PMAD during the COVID-19 pandemic [39]; COVID-19 has exacerbated underlying disparities in care for perinatal individuals [40,41]. These findings and policy guidance could influence outcomes for these high-cost, high-risk individuals.

## DISCUSSION

Depression and anxiety are among the most common conditions complicating pregnancy and the postpartum period. They can have long-lasting, negative ramifications for perinatal individuals, children, and families—a multigenerational burden. Worldwide, researchers recognize “there is no health without perinatal mental health” [42]. PMAD is associated with adverse pregnancy outcomes and compromised parenting in perinatal individuals, and impaired affect, behavior regulation, and insecure attachment in children [43,44]. Clinicians detect a small minority of individuals with PMAD; few of these individuals receive care and fewer receive adequate treatment or achieve remission [2,45]. A meta-analysis coined the phrase “*Perinatal Depression Treatment Cascade*” [2], noting treatment decrements at each branch of the cascade.

Depression prevalence in women peaks during reproductive years [46], yet pregnancy neither protects nor exacerbates depression risk [47]. This fact counters traditional notions that pregnancy protects perinatal individuals against depression [6,48,49]. Each year, >650,000 infants are born to individuals with perinatal depression (PND), the most underdiagnosed obstetric complication in the US [2,19]. PND encompasses depression during pregnancy to one year post-delivery. Individuals with PND and their babies have significantly greater healthcare costs due to higher premature birth rates, decreased well-child visits, greater use of inpatient and ED services, and longer peripartum lengths of stay [50,51]. Lifetime costs of not treating PND exceed $15 billion annually in the US, with short- and long-term negative consequences for birthing parent and child [2].

Most research and clinical interventions for perinatal MH conditions focus on PND, yet perinatal anxiety (PA) is also prevalent and serious, with similar negative outcomes and high costs. Some studies indicate PA prevalence exceeds PND prevalence [52]; a recent meta-analysis estimated that the prevalence of perinatal anxiety is 20.7% [53]. Comorbid antenatal depression and anxiety is the rule, not the exception. One study found that the prevalence of a clinical diagnosis of any anxiety disorder alongside depression reached 9.3% antenatally and 4.2% postnatally [54]. Due to overlap between the two conditions, it may be more useful to consider PND and PA in a spectrum of perinatal psychiatric illness, or PMAD [55,56]. PMAD is prevalent and persistent, particularly given almost no attention to PA.

### Outcomes

As PMAD rise in the US, individuals with PMAD have worse outcomes and higher cost delivery hospitalizations, with widening disparities in the burden of many common, chronic conditions (including MH disorders) by race and ethnicity, low income, and among rural populations [10,57–59]. Using 2006–2015 National Inpatient Sample (NIS) data [11], we compared PMAD and serious mental illness (SMI) among delivering individuals over time. Among 2006–2015 deliveries, we found worse delivery outcomes and higher levels of healthcare use and expenditures among perinatal individuals with versus without MH disorders. We also found a higher incidence of SMM among commercially insured Black perinatal individuals with MH conditions compared to non-Hispanic White perinatal individuals with MH conditions [33].

Additional data document disparities in PMAD prevalence and associated treatment and outcomes, including overlapping disparities (e.g., minority and low-income, rural and low-income). For example, Black and Hispanic perinatal individuals have lower rates of initiating treatment, receiving follow-up care, and maintaining antidepressant use than White perinatal individuals [24]. Between 5% and 25% of pregnant individuals have depression; lower-income pregnant individuals suffer at a higher rate (40–60%), with more severe symptoms and episodes [19], and lower rates of treatment engagement and retention [20]. Price-sensitive individuals may forgo needed care [60,61], leading to poor management of MH needs, and negative repercussions before and after delivery. Perinatal individuals living in rural areas are twice as likely to experience PND than urban peers [21,22]. Rural perinatal individuals may experience more treatment barriers, such as lack of local providers and travel burdens to distant care [62].

PMAD also contributes to rising rates of maternal morbidity in the US, particularly in the postpartum period [59]. Psychiatric illness is the third most common indication (after infection and hypertension) and the most common diagnosis for postpartum readmission, accounting for 7.7% of readmissions [63]. Readmission within 30 days of delivery increased between 2004 and 2011; low-income and Black perinatal individuals had higher rates of all-cause postpartum readmissions, and PMAD influences this trend [63]. SMM is closely linked to high rates of maternal mortality among Black and Hispanic perinatal individuals [8,9,64,65]. MH conditions and suicide are leading causes of pregnancy-related death [7,8,66,67], highlighted in a longitudinal study of 14 maternal mortality committees [68]. Additionally, untreated PMAD increases likelihood of adverse birth outcomes such as low birth weight, preterm birth, decreased fetal growth [69–71].

Working to address untreated PMAD is especially timely because the COVID-19 pandemic has had a substantial negative impact on perinatal individuals, exacerbating underlying disparities in care and outcomes. Evidence suggests a staggering burden of COVID-19 on perinatal individuals. One study found the prevalence of postpartum depression 15% pre-pandemic and 41% during the pandemic; postpartum anxiety rose from 29% pre-pandemic to 72% during the pandemic [39]. Additional studies have confirmed that the pandemic has had a significant impact on the mental health of pregnant and postpartum individuals due to factors such as maternal fear of vertical transmission of the virus to their infants, limited accessibility of antenatal care resources, and lack of social support [72]. Since the risk for pandemic-related PMAD appeared greatest during the postpartum period, increased screening and intervention during this period may remain important [73]. Perinatal individuals of color reported significantly higher levels of pandemic-related stress than their White counterparts [40]. We anticipate future published data revealing the disparities within negative impacts of the virus on perinatal individuals with PMAD, including increased morbidity and mortality.

Given the significant negative impact of PMAD on outcomes and availability of effective PMAD treatment, reducing PMAD should improve outcomes.

### Screening and Treatment

Clinical guidelines recommend depression and anxiety screening throughout the perinatal period with validated screening tools [4,19,74]. Screening without follow-up is ineffective [4], and perinatal individuals may face multiple individual and community barriers to accessing PMAD care [75,76]. Barriers include stigma, lack of obstetric provider training, lack of resources, and limited access to MH treatment. Facilitators include empowering perinatal individuals during health care provider interactions, obstetric provider and staff training, standardized screening and referral processes, and improved MH resources. A promising recent meta-analysis found evidence that postpartum individuals undergoing screening programs for depression reported improved depressive and anxiety symptoms [77]; however, researchers should further study and characterize the relationship between screening and PMAD outcomes. In 2022, the Healthcare Effectiveness Data and Information Set reported average performance rates for its two perinatal behavioral health measures: the Prenatal Depression Screening and Follow-Up (PND-E) and the Postpartum Depression Screening and Follow-Up (PDS-E) [78]. In the case of PND-E, the average rates of screening and follow-up were 7.0%–8.8% and 50.2%–56.0%, respectively, from 2020–2021 for individuals with commercial insurance, while the average rates of screening and follow-up were 14.0%–15.7% and 52.1%–49.7%, respectively, from 2020–2021 for individuals with Medicaid. In the case of PDS-E, the average rates of screening and follow up were 8.3%–11.1% and 55.6%–64.5%, respectively, from 2020–2021 for individuals with commercial insurance, while the average rate of screening and follow-up were 13.8%–16.5% and 55.458.6%, respectively, from 2020–2021 for individuals with Medicaid. Clinical, program, and system modifications could optimize PMAD care [76].

Treatment for PMAD is a key modifiable area to increase obstetrical care value [18]. Birth is one of the most common reasons for healthcare use in the US and a top expenditure for payers every year [18,79]. Since 2000, birth-related payments have increased without significant improvements in perinatal outcomes [80,81]. Between 2004 and 2010, commercial maternity care payments increased by >50%, with a four-fold increase in out-of-pocket payments [82]. Annual payments associated with pregnancy, birth, and postpartum care totaled $87 billion. Implementing MH treatment for high risk pregnant individuals could decrease costs from preterm birth by 10%–25%, decreasing total costs of US maternity healthcare spending by 2% [83].

### Costs of Illness

Given high costs of obstetric care and of PMAD for both perinatal individuals and children and the cost-effectiveness of PMAD treatment, targeting this expensive period and vulnerable population is a public policy priority. The present value of total lifetime costs of perinatal depression (anxiety) was $121,165 ($55,698) per perinatal individual in 2012–2013 in the UK [12]. Prevalence estimates of the respective cost of PMAD combined was $13,600 per pregnant individual giving birth; with aggregated costs of $10.6 billion. US estimates including PND (but not PA) reached $15 billion per year [2]. This study identified incremental cost effectiveness ratios of $13,857 per quality-adjusted life year (QALY, a generic measure of disease burden, including both quality and quantity of life lived) gained and $10,182 per remission achieved [84], well below recommended cutoff thresholds for cost-effectiveness of $50,000–$100,000 per QALY [85]. Another study found that the societal costs of untreated PMAD in the US reached $14.2 billion in 2017 [86].

### Opportunities for Improvement

#### Leveraging prenatal care.

Pregnant individuals are likely to be engaged with the healthcare system during pregnancy, providing an opportunity to address PMAD, yet the impact of community factors on treatment is unknown. Prenatal care involves regular contact with the healthcare system (with >50 million visits annually), consisting of approximately 12 visits per person (compared to 2–4 annual visits among non-pregnant reproductive-aged individuals) [87,88]. Regular contact provides opportunities to assess fetal development and maternal wellbeing. Variation and disparities in how MH needs are addressed in perinatal visits remains unknown. How community factors such as availability of providers and facilities influences MH utilization associated with PMAD is undetermined. Our team aims to determine the contributions of such community-level characteristics to perinatal healthcare utilization and delivery outcomes among women with PMAD.

#### Working toward cohesive national strategy.

There is no cohesive national strategy to address PMAD. Depending on where a person resides, the approach to PMAD identification, diagnosis, and care may vary widely. Treatment may include multiple providers (family or internal medicine, OB/GYN, pediatrics, specialty MH). In 2016, the Centers for Medicare and Medicaid Services (CMS) issued policy guidance to clarify that state Medicaid agencies may pay for maternal depression screening during a well-child visit as risk assessment for the child under the Early and Periodic Screening, Diagnostic and Treatment (EPSDT) Medicaid benefit [89]. In 2016, the US Preventive Services Task Force (USPSTF) recommended screening pregnant and postpartum individuals for depression [74]; in 2019 USPSTF recommended counseling for individuals at risk for perinatal depression [90]. In 2013, only 10 states covered maternal depression screenings under Medicaid [91–93]. By 2017, 37 states recommended, required, or allowed maternal depression screening as part of a well-child visit, 5 states implemented reimbursement or other guidelines, 4 states had maternal depression screening performance measures, 13 states had none [94]. Whether and how such policies influence access to care or outcomes remains unknown. Pediatrician-reported barriers to offering such screening during well-child visits represent potential targets for improvement and include lack of time and being unfamiliar with mental health resources [95].

*“Increase the proportion of women who get screened for postpartum depression”* is a “developmental measure” in *Healthy People 2030*, the federal government’s goals for improving Americans’ health [96], because there are no national data for this measure (these data come from PRAMS). There has been little rigorous national examination of healthcare utilization of individuals with PMAD and factors associated with its variation. Hence, a nationwide de facto “natural experiment” exists, as state approaches to managing PMAD vary.

Our team is examining patterns of PMAD treatment and outcomes in publicly and privately insured populations, which will provide a comprehensive picture of populations with the greatest unmet needs and associated morbidity. Studying predictors and outcomes of state variation has significance and timeliness now because PMAD rates are rising [11], and negative birth outcomes, such as SMM [97], are increasing. PMAD contributes to these outcomes, as MH conditions are among the top five leading causes associated with pregnancy-related deaths [8].

#### Leaning into evidence-based policymaking.

Few states engage in evidence-based policymaking (EBP). In 2014, the Pew Charitable Trusts and the MacArthur Foundation published “Evidence-Based Policymaking,” including five key steps in EBP [98]. Their 50-state analysis of state human service policymaking evaluated how states engage in EBP. They found that only five states were fully engaged in formulating EBP [99]. When the Substance Abuse and Mental Health Services Administration (SAMHSA) required recipients of community MH grants to report the prevalence of 10 evidence-based programs, recipients placed greater value on such programs, and significantly increased the number of evidence-based treatments offered to MH clients. In 2015, the Council on Patient Safety in Women’s Health Care developed a patient safety bundle to address maternal MH (readiness, recognition and prevention, response, reporting and systems learning) [100]. How this bundle has been implemented is unknown. The frequency of maternal depression screening, and positive screens leading to evidence-based treatment, is also unknown. Our team aims to collaborate with a panel of experts who work closely with policymakers to support evidence-based policymaking for PMAD.

#### Generating rankings to facilitate change.

Rankings are an important tool of public health surveillance. The Institute of Medicine report, *The Future of Public Health*, identifies assessment (including surveillance, identifying needs, collecting, and interpreting data) as one of three core public health functions (in addition to policy development and assurance). The report argues that states have “primary responsibility for the well-being…of their citizens” [101]. America’s Health Rankings [102] has monitored health in all 50 states since 1990 [103]. Rankings allow public health leaders to target resources and advocate for investment [103]. A state health official study found that rankings provided useful data for problem identification, and called for them to be “more actionable” and help identify best practices [104]. Rankings generate media coverage, educate legislators and the community, and identify program targets [105]. Starting in 2018, the rankings included proportion of individuals delivering a live birth who experience PPD [106,107].

For Association of State and Territorial Health Officials’ (ASTHO) members, rankings are a driver for change. ASTHO researched how rankings are used to improve health nationally. For example, Louisiana (ranked 50 in 2016) used its rankings to transform its state health system in at least two relevant ways: 1) it developed a “Best Babies Zone” to reduce infant mortality and racial disparities in birth outcomes by creating neighborhood-level heat maps of vital records birth data to track low birth weights; and 2) it provides incentives for improving a quality measure focused on pre-term birth [108,109]. ASTHO also highlighted 2019 state legislative activity focused on maternal mental health screening, a key area amenable to policy reform [110].

As part of our work, we plan to develop such state-level profiles focused on PMAD.

## CONCLUSIONS

PMAD is a burdensome and costly disorder with known effective treatments. Where a person lives determines the nature and quality of PMAD care. Patient and community factors likely influence whether individuals with PMAD, regardless of insurance type, access effective treatment and avoid negative outcomes. State and national policies regarding PMAD screening and treatment are evolving [92,93], yet it is unclear how these policies have influenced practice and outcomes. Given that relatively few states engage in EBP, to meet the growing public health crisis of negative perinatal outcomes in the community, it is critical that states understand the extent of and populations at high risk for poor outcomes and identify strategies to implement evidence-based PMAD care, tailored to address disparities in access, treatment, and outcomes.

## Data Availability

No data were generated from the study.
